# Supplementary material for: Hypnosis-Assisted Awake Craniotomy for Eloquent Brain Tumors: Advantages and Pitfalls
Source: Cancers (Basel). 2024 May 5;16(9):1784. doi: 10.3390/cancers16091784 (PMC11083963; doi:10.3390/cancers16091784)
Supplement: Supplementary file 1 [file cancers-16-01784-s001.zip › cancers-2983290-supplementary.pdf]

A faire remplir EN POST-OPERATOIRE

Questionnaire de satisfaction destiné aux patients bénéficiant d'une chirurgie éveillée (avec/sans hypnose)

Au moyen de ce questionnaire, nous aimerions connaître votre **niveau de satisfaction** dans les différentes phases de votre prise en charge, en pré-opératoire, pendant l'opération (per-opératoire) et en post-opératoire.

Pour chaque question, choisissez la réponse qui correspond le mieux à ce que vous ressentez à l'heure actuelle en inscrivant "X" dans la bonne case.

5 niveaux de satisfaction

1 = PAS DU TOUT
2 = PEU
3 = MOYENNEMENT
4 = PLUTÔT
5 = TRES

AVANT l'intervention :	1	2	3	4	5
1.Est-ce que les explications **du neurochirurgien** concernant la chirurgie ont été claires et suffisantes ?					
2.Est-ce que les explications de l'**anesthésiste** concernant la chirurgie ont été claires et suffisantes ?					
3.Est-ce que les explications de la **neuropsychologue** concernant la chirurgie ont été claires et suffisantes ?					
4.Avez-vous été suffisamment préparé(e) en terme d'information sur le déroulement pratique de la chirurgie ?					
5.Avez-vous été suffisamment informé(e) des risques / des séquelles de la chirurgie ?					

5 niveaux de satisfaction

1 = PAS DU TOUT
2 = PEU
3 = MOYENNEMENT
4 = PLUTÔT
5 = TRES

PENDANT l'intervention :	1	2	3	4	5
6.Etait-ce difficile de répondre aux tests pendant la chirurgie ?					
7.Parveniez-vous à voir distinctement les images à dénommer ?					
8.Que pensez-vous de la durée des tests, était-t-elle trop longue ?					
9.Vous êtes-vous senti(e) bien entouré(e) durant l'intervention par les différents professionnels (chirurgien, anesthésiste, neuropsychologue)					
10.La position dans laquelle vous vous trouviez pendant l'intervention était-elle confortable ?					

- 11. Avez-vous ressenti des douleurs pendant la chirurgie ?

  ☐ Pose de cathéter veineuse/artériel (11a)
  ☐ Injections of local anesthésie au niveau du crâne (11b)
  ☐ Mise en place de la têtière (11c)
  ☐ Incision (11d)
  ☐ Réalisation des trous de trépan (11e)
  ☐ Fermeture (11f)

  -Autres (11g) :

- 12. Avez-vous des souvenirs de la chirurgie ? Aucun souvenir (0) ☐ Quelques souvenirs (1) ☐ Je me souviens de tout (2) ☐
    - 12a. Si oui, les souvenirs sont-ils globalement positifs ou négatifs ? (Souligner la réponse qui convient)
    - 12b. Quels souvenirs gardez-vous en quelque mots ?
- 13. A-t-on utilisé l'hypnose pendant la chirurgie ? Oui ou NON (soulignez la bonne réponse)
- 14. Si vous avez utilisé l'hypnose merci de répondre aux prochaines questions :
    - 14a. Quel(s) moment(s) était(ent) agréable(s) pendant la chirurgie ?
    - 14b. Vous rappelez-vous de l'histoire racontée pendant l'hypnose ? OUI ☐ NON ☐
    - 14c. Si vous vous rappelez de l'histoire, comment la décririez-vous : relaxante, amusante, agréable, absurde, angoissante (souligner une ou plusieurs réponses)
    - 14d. Si vous vous rappelez de l'histoire, est-ce que vous vous êtes senti(e) en phase/connecté(e) à cette histoire impliqué(e) prise par l'histoire ? OUI ☐ NON ☐
    - 14e. Avez-vous conservé la notion du temps pendant l'hypnose ? OUI ☐ NON ☐
    - 14f. Vous rappelez-vous de la fin de la chirurgie, après la fin des tests neuropsychologiques ? OUI ☐ NON ☐

5 niveaux de satisfaction

1 = PAS DU TOUT
2 = PEU
3 = MOYENNEMENT
4 = PLUTÔT
5 = TRES

APRES l’intervention :	1	2	3	4	5
15.Vous sentez-vous soulagé(e) ?					
16.Vous sentez-vous choqué(e) par la chirurgie ?					
17.Pensez-vous avoir besoin d’un soutien psychologique après avoir vécu cette chirurgie éveillée ?					

- 18. Qu’a changé cette opération dans votre vie en quelques mots ?

____

____

- 19. Si vous deviez bénéficier d’une nouvelle chirurgie du cerveau, accepteriez-vous une seconde fois cette expérience de la chirurgie éveillée ?

  OUI ☐ NON ☐ Si oui, avec ou sans hypnose ? (Souligner la réponse qui convient)
